# Gut Microbiota According to the Metabolome

**DOI:** 10.3390/nu15224768

**Published:** 2023-11-13

**Authors:** Emidio Scarpellini, Emanuele Rinninella

**Affiliations:** 1 Translationeel Onderzoek van Gastro-Enterologische Aandoeningen (T.A.R.G.I.D.), Gasthuisberg University 11 Hospital, KU Leuven, Herestraat 49, 3000 Leuven, Belgium; 2 Research and Training Center in Human Nutrition, Catholic University of Sacred Heart, 00168 Rome, Italy; emanuele.rinninella@unicatt.it

The human gut microbiota is an ecosystem harboring trillions of microorganisms, encompassing bacteria, viruses, archaea, fungi, and protozoa [1]. Altogether, these organisms participate in absorptive, metabolic, and immune functions in our intestines [2]. Changes in the gut microbiota composition occur in gastrointestinal and extra-gastrointestinal diseases [3,4]. Some metabolic diseases include diabetes and insulin resistance, obesity, hypertension, dyslipidemia, and metabolic-associated fatty liver disease (namely, MAFLD) [5]. The demodulation of the gut microbiota is called “dysbiosis” and can be caused by antibiotic and pre- and probiotic usage [6,7]. These living organisms beneficially affect human health [7]. Traditional culture-based techniques and more recent metagenomic assessments have allowed the use of gut dysbiosis as a disease biomarker to study treatment responses [8]. 

However, the products of microbial metabolism can significantly affect human health, and research efforts must focus on their study and characterization [8]. The birth of metabolomics, namely the profiling of metabolites in biofluids, cells, and tissues, has paved the road toward a deeper and better understanding of human metabolic processes [9]. By definition, metabolites include substrates, the products of the metabolism of cells, and their crucial functions (e.g., energy production and storage, signal transduction, and apoptosis) [10]. Metabolites are usually produced by the human body, but they can also be produced by microorganisms, xenobiotics, and dietary sources [11]. Metabolites’ functions range from the regulation of epigenetic mechanisms to the maintenance of the pluripotency of embryonic stem cells [12,13]. In addition, metabolites, such as ATP, acetyl-CoA, NAD+, and Sadenosyl methionine (SAM), can also regulate the post-translational modification of protein activity [14]. Metabolic products can also maintain and/or affect the cellular/extracellular environment of production such as in cancer cells and tissues [15].

Thus, the knowledge of metabolites and their entire composition, namely “metabolome”, is crucial for maintaining health and managing disease in humans. Metabolomics is based on two main methodologies that involve metabolite recovery and identification: untargeted and targeted mass spectrometry-based metabolomics. The first method measures the metabolites present in an extracted sample without knowledge of the metabolomic mechanisms behind it. The second one provides higher sensitivity and selectivity vs. untargeted methods because metabolites are analyzed according to hypothesized pathways. Indeed, integrating targeted analysis helps to validate the results from untargeted techniques [16]. The most recent informatics, stable isotope-assisted metabolomics, and big data integrative analysis across different omics (namely, genomics, epigenomics, proteomics, and transcriptomics) allow orthogonal metabolomics constructs that are the basis for metabolic processes understanding. For example, this orthogonal approach has shown the role of bacterial biofilms in cancer pathophysiology and the metabolic regulation of cell pluripotency. It has provided data on new metabolic treatments for cardiovascular, pancreatic beta-cell dysfunction, cancer, and ischemia-reperfusion injuries [17]. 

The products of gut microbial metabolism are post-biotics. The fine interaction between the intestinal metabolome and humans is involved in disease pathogenesis and has emerging strong therapeutic implications. 

In this Special Issue, we want to highlight the effect of gut microbial products on human health, with a special focus on probiotics’ metabolome as a promising treatment for gastrointestinal and systemic diseases.

## Data Availability

Data reported in this Editorial are available online on PubMed and main gastrointestinal and microbiological international meetings’ database.
